# Prognostic Value of Right Ventricular Strains Using Novel Three-Dimensional Analytical Software in Patients With Cardiac Disease

**DOI:** 10.3389/fcvm.2022.837584

**Published:** 2022-02-25

**Authors:** Tetsuji Kitano, Attila Kovács, Yosuke Nabeshima, Márton Tokodi, Alexandra Fábián, Bálint Károly Lakatos, Masaaki Takeuchi

**Affiliations:** ^1^Department of Cardiology and Nephrology, Wakamatsu Hospital of the University of Occupational and Environmental Health, Kitakyushu, Japan; ^2^Heart and Vascular Center, Semmelweis University, Budapest, Hungary; ^3^Second Department of Internal Medicine, School of Medicine, University of Occupational and Environmental Health, Kitakyushu, Japan; ^4^Department of Laboratory and Transfusion Medicine, University of Occupational and Environmental Health Hospital, Kitakyushu, Japan

**Keywords:** right ventricular (RV), right ventricular ejection fraction, three-dimensional strain (3D strain), prognosis, ReVISION, cardiac disease

## Abstract

**Background:**

Right ventricular (RV) three-dimensional (3D) strains can be measured using novel 3D RV analytical software (ReVISION). Our objective was to investigate the prognostic value of RV 3D strains.

**Methods:**

We retrospectively selected patients who underwent both 3D echocardiography (3DE) and cardiac magnetic resonance from January 2014 to October 2020. 3DE datasets were analyzed with 3D speckle tracking software and the ReVISION software. The primary end point was a composite of cardiac events, including cardiac death, heart failure hospitalization, or ventricular tachyarrhythmia.

**Results:**

341 patients were included in this analysis. During a median of 20 months of follow-up, 49 patients reached a composite of cardiac events. In univariate analysis, 3D RV ejection fraction (RVEF) and three 3D strain values [RV global circumferential strain (3D RVGCS), RV global longitudinal strain (3D RVGLS), and RV global area strain (3D RVGAS)] were significantly associated with cardiac death, ventricular tachyarrhythmia, or heart failure hospitalization (Hazard ratio: 0.88 to 0.93, *p* < 0.05). Multivariate analysis revealed that 3D RVEF, three 3D strain values were significantly associated with cardiac events after adjusting for age, chronic kidney disease, and left ventricular systolic/diastolic parameters. Kaplan-Meier survival curves showed that 3D RVEF of 45% and median values of 3D RVGCS, 3D RVGLS, and 3D RVGAS stratified a higher risk for survival rates. Classification and regression tree analysis, including 22 clinical and echocardiographic parameters, selected 3D RVEF (cut-off value: 34.5%) first, followed by diastolic blood pressure (cut-off value: 53 mmHg) and 3D RVGAS (cut-off value: 32.4%) for stratifying two high-risk group, one intermediate-risk group, and one low-risk group.

**Conclusions:**

RV 3D strain had an equivalent prognostic value compared with 3D RVEF. Combining these parameters with 3D RVEF may allow more detailed stratification of patient's prognosis in a wide array of cardiac diseases.

## Introduction

For the last decade, the right ventricle has gained increasing attention for morphological and functional assessment. Although tricuspid annular plane systolic excursion (TAPSE), right ventricular (RV) fractional area change, systolic tricuspid annular velocity (RVs'), and longitudinal strains are commonly used for assessment of RV function with two-dimensional (2D) and tissue Doppler echocardiography, these measurements do not cover all aspects of RV function and 2D echocardiographic assessment of RV function has several limitations due to the complex morphology of the right ventricle. Three-dimensional echocardiography (3DE) provides accurate and reproducible values of RV volumes and ejection fraction (RVEF) (1, 2) and current guidelines recommend its use to examiners who are familiar with 3DE (3). We have previously reported that RVEF by 3DE is useful for predicting future prognosis in various cardiac diseases (4–6).

The ReVISION (Right VentrIcular Separate wall motion quantificatiON) software was recently introduced, enabling comprehensive 3D echocardiographic analysis of the right ventricle (7). With this method, strain values such as 3D RV global circumferential strain (RVGCS), 3D RV global longitudinal strain (RVGLS), and 3D RV global area strain (RVGAS) could also be calculated from 3DE datasets. Area strain was defined as the percentage change of the regional area of the endocardium, which can be regarded as the product of both longitudinal strain and circumferential strain. Although reference values of 3D RVGCS, 3D RVGLS, and 3D RVGAS were established using ReVISION software (8), there are no studies indicating whether 3D RV strains have prognostic value over 3DE-derived RVEF in patients with cardiac disease.

We hypothesized that 3D RV strains would provide incremental prognostic information over 3D RVEF. Accordingly, we sought to investigate the prognostic value of RV 3D strains in a diverse group of subjects.

## Materials and Methods

### Study Subjects

This was a single-center, retrospective, observational study. Using a cardiac magnetic resonance (CMR) database, we retrospectively selected patients who underwent both CMR and transthoracic echocardiography on the same day at the University of Occupational and Environmental Health Hospital from January 2014 to October 2020. Exclusion criteria were repeat examinations, age <20 years, patients with <30 days of follow-up, patients without echocardiographic datasets, and patients with extremely poor image quality. Clinical characteristics such as anthropometric data, risk factors, and medication information on the day of the echocardiography were collected. This study protocol was approved by the local Ethics Committee of the University of Occupational and Environmental Health (IRB No: UOEHCRB20-181). The requirement for written informed consent was waived because of the retrospective nature of the study.

### Echocardiographic Acquisition

Comprehensive transthoracic two-dimensional and Doppler echocardiographic examination were performed using a commercially available ultrasound system (iE33 or Epic7G, Philips Medical System, Andover, Massachusetts; Vivid E95, GE Healthcare, Horten, Norway).

3DE was performed according to guidelines of the American Society of Echocardiography (ASE) using an iE33, Epic7G, or Vivid E95 equipped with a 3DE transducer (X5-1, Philips Medical System, Andover, Massachusetts; 4V or 4Vc, GE Healthcare, Horten, Norway) (3). 3DE datasets that focused on the left heart chamber were acquired from the apical approach in one- or multi-beat acquisition mode. In addition, 3DE datasets that focused on the right heart chamber were acquired from more lateral transducer positions. In order to increase the volume rate, the width of the image sector size was reduced as narrow as possible, keeping orthogonal 2D echocardiographic images encompassing the entire right ventricle. Datasets were transferred to a separate workstation for off-line analysis.

### Echocardiographic Analysis

Echocardiographic analysis was performed by an experienced examiner (MT). Traditional transthoracic 2D and Doppler echocardiographic parameters were measured according to current guidelines of the ASE and the European Association of Cardiovascular Imaging (9).

Image quality of 3DE datasets was subjectively evaluated by visibility of the endocardium in 2D echocardiography images extracted from 3DE datasets (apical four-chamber, two-chamber, and long-axis view for the left ventricle, and in apical four-chamber view, coronal view, and basal short-axis view for the right ventricle). Each view was divided into six segments, and each segment was scored as 0 (no endocardial visualization), 0.5 (partial endocardial visualization during one cardiac cycle), or 1 (complete endocardial visualization throughout one cardiac cycle). These scores were summed to calculate the total image score (range: 0–18). Results were categorized as good (defined as a total score ≥ 16), fair (13 ≤ score < 16), poor (10 ≤ score < 13), or extremely poor (score < 10) (5).

For 3DE analysis of the left ventricle (LV), 3DE datasets that focused on the left heart chamber were analyzed using vendor-independent 3D speckle tracking software (4D LV analysis, TomTec Imaging System, Unterschleissheim, Germany). The software automatically extracted apical four-chamber, two-chamber, long-axis, and short-axis views from 3DE datasets and detected endocardial surface at LV end-diastole. Endocardial borders at LV end-diastole and end-systole were manually corrected as required. After endocardial boundaries were determined, the software conducted 3D speckle tracking analysis over the entire cardiac cycle and automatically measured LV end-diastolic volume (LVEDV), LV end-systolic volume (LVESV), LV ejection fraction (LVEF), and LV global longitudinal strain (LVGLS).

**Figure 1:**
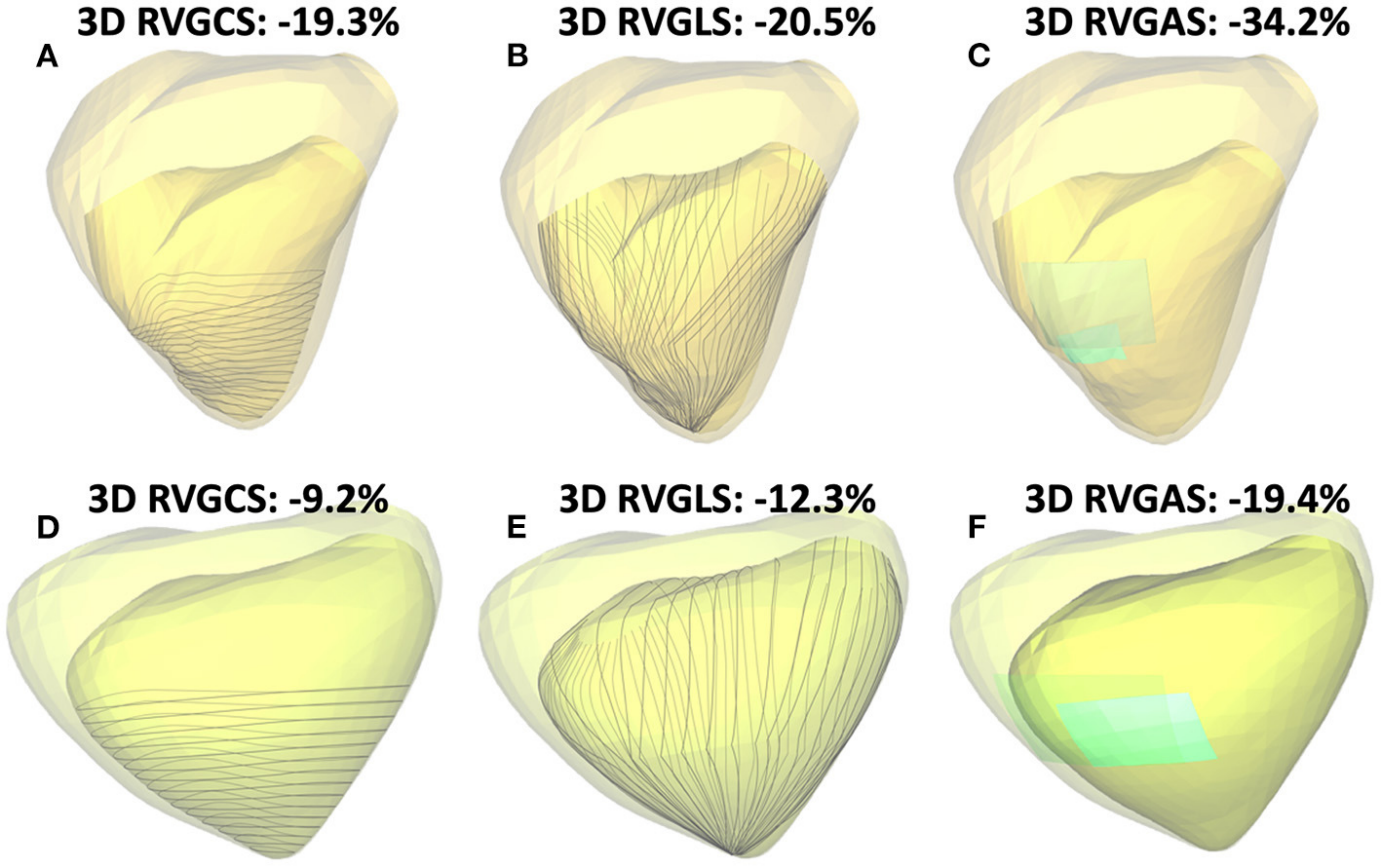
Two representative cases of three-dimensional (3D) right ventricular (RV) analysis using ReVISION software: One case of normal RV function (3D RVEF: 49%, **A–C**) and another case of severe RV dysfunction (3D RVEF: 28%, **D–F**). Transparent light-yellow and light-green represents the end-diastolic endocardial boundary, and darker yellow and darker green represents the end-systolic endocardial boundary. Gray lines indicate contours used for 3D RV global circumferential strain (RVGCS) **(A,D)**, and 3D RV global longitudinal strain (RVGLS) **(B,E)** assessment, respectively. Light-blue segmental areas and their change from end-diastole to end-systole represent the rationale behind area strain calculations [3D RV global area strain (RVGAS) is calculated using entire RV endocardial areas, **C,F**].

For RV analysis, we used commercially available 3D speckle tracking software (4D RV function 3, TomTec Imaging system, Unterschleissheim, Germany) and ReVISION software (Argus Cognitive, Inc., Lebanon, New Hampshire, USA, www.revisionmethod.com). First, 3D models of the right ventricle were created with 4D RV function 3. After selecting a 3DE dataset that focused on the right heart chamber, the software automatically extracted two orthogonal views of the left and right ventricles from the 3DE datasets. After aligning each view and determining several anatomic landmarks (the LV apex and the middle of the mitral annulus, the RV apex and the middle of the tricuspid annulus), RV endocardial boundaries were automatically drawn by the software. RV endocardial borders at end-diastole were manually corrected as required. The software performed speckle tracking analysis resulting in calculation of RV volumes and RVEF. 3D RV models were exported throughout the cardiac cycle as a 3D object file series and transferred to ReVISION software. The detailed analytical method of ReVISION software has been described elsewhere (10). Briefly, the software calculates conventional volumetric parameters, i.e., RV end-diastolic volume (RVEDV), RV end-systolic volume (RVESV), and RV ejection fraction (RVEF). To evaluate 3D RVGCS, 15 circumferential contours were created by slicing the 3D model with horizontal planes at equal distances along the longitudinal axis and were tracked throughout the cardiac cycle. The length of the latitudes (C) can be calculated by subdividing the curve and calculating it as the sum of the straight lines. RVGCS can be calculated by multiplying the sum of (C^end−systole^-C^end−diastole^/C^end−diastole^) of the 15 latitudes by 100. To evaluate 3D RVGLS, 45 longitudinally oriented contours were generated by connecting the RV apex and predefined points of the RV base and were tracked throughout the cardiac cycle. The length of the longitude (L) can be calculated as the sum of the apex-middle section of the RV and middle section of the RV-RV base geodesic distances. RVGLS can be calculated by multiplying the sum of (L^end−systole^-L^end−diastole^/L^end−diastole^) of 45 longitudes by 100. RVGAS was calculated from the relative change of the endocardial surface between end-diastole and end-systole. The surface is divided into a triangular mesh and its surface area (A) is calculated. The sum of the surface areas at the end of systole (A^end−systole^) and at the end of diastole (A^end−diastole^) is calculated, and the RVGAS is calculated by multiplying (A^end−systole^-A^end−diastole^/A^end−diastole^) by 100 (Figure 1). RV volumes, RVEF and 3D RV strains (RVGCS, RVGLS, and RVGAS) with ReVISION software were used for the main analysis, while RV volumes and RVEF derived from TomTec software (4D RV function 3) were also presented and used for some analysis.

### Follow-Up

Follow-up information was obtained by two researchers (TK, YN), who were not involved in the echocardiographic analysis. Patients were followed up regularly in the outpatient clinic. For patients attending our hospital, prognostic information, such as whether and when a cardiac event occurred, was obtained from the attending physician or the electronic medical record. For patients undergoing treatment at other hospitals, we called the patient at home. If consent was obtained, we asked the patient or family about their current health status and whether and when a cardiac event had occurred. The day of echocardiography was defined as day 0, and final follow-up data were obtained in February 2021. The primary endpoint was a composite of cardiac events, including cardiac death, sustained ventricular tachyarrhythmia, or heart failure (HF) hospitalization. If patient developed multiple events, we selected hardest one as following order (HF hospitalization < sustained ventricular tachyarrhythmia < cardiac death). The secondary endpoint was HF hospitalization.

### Reproducibility Analysis

Intra-observer variability of 3D RV volumes and 3D RVEF by 4D RV function 3 was evaluated by repeating measurements taken by the examiner on 35 randomly selected patients at interval of at least one-month, inter-observer variability was evaluated by a second examiner taking these measurements on the same 35 patients.

### Statistical Analysis

Commercially available statistical software was used for statistical analysis (JMP Version 14.3.0, SAS Institute, Cary, North Carolina, USA; R Version 4.1.2, The R foundation for Statistical Computing, Vienna). Continuous variables were represented as medians and interquartile ranges (IQR). Categorical variables were expressed as frequencies or percentages. Comparisons between the two groups were analyzed using *t*-tests or Mann-Whitney U tests for continuous variables, and Fisher's exact test or the chi-square test for categorical variables. A correlation analysis was performed with the r value of the Spearman rank correlation coefficient. Numbers needed to treat (NNTs) were calculated as indicators of effect size (11). Survival time analysis was evaluated using the Kaplan-Meier method, and differences between groups were determined using the log-rank test. A cox proportional hazards model was built to calculate hazard ratio (HR) and 95% confidence interval (CI). The nested regression model was used to assess the incremental prognostic value. A decision tree model was created using classification and regression tree (CART) analysis, which divided patients into binary groups with the highest outcome contrasts and also estimated appropriate cutoff values to predict time-to-event outcomes (12).

## Results

**Figure 2:**
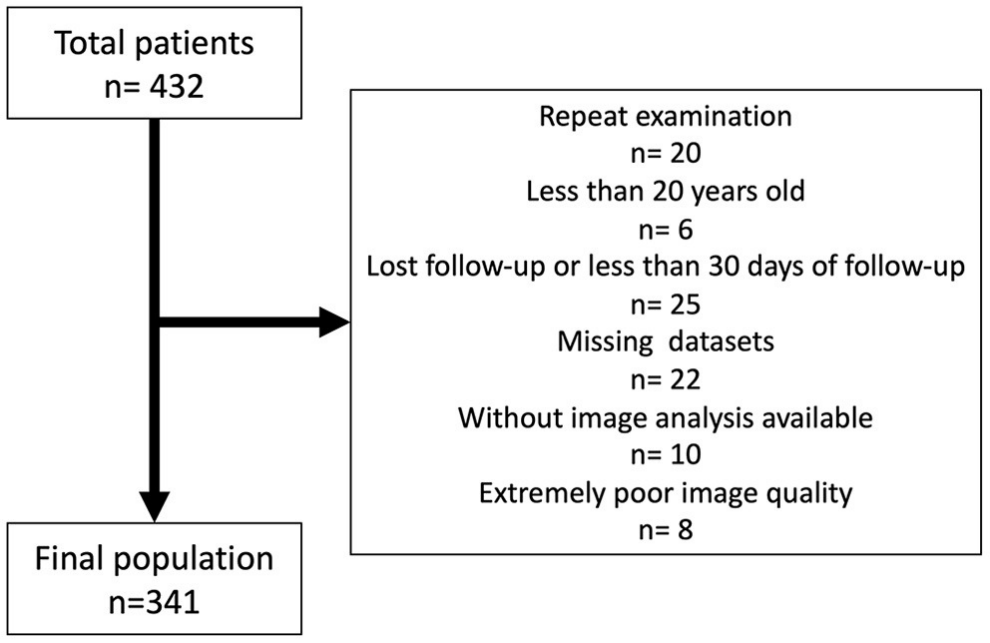
Flow chart of the study population.

Of 432 patients enrolled in the CMR database from January 2014 to October 2020, 341 patients were included as a final study population (Figure 2). Feasibilities of 3D LV and RV analysis were 99% (340/341), and 100% (341/341). Image quality of the left ventricle was good in 20% (68/341), fair in 47% (160/341), and poor in 33% (113/341), respectively. Corresponding values of the right ventricle were 14% (45/341), 42% (144/341), and 44% (152/341) respectively. The median volume rate was 23 Hz (IQR: 20–27 Hz, range: 15–58 Hz). Table 1 shows clinical characteristics of study subjects.

**Table 1:** Clinical characteristics in the study population.

**Number**	***n* = 341**
Age (year)	68 (58, 76)
Male	226 (66)
Sinus	310 (91)
AFib	31 (9)
**Risk factor**	
HT	191 (56)
DM	101 (30)
HL	149 (44)
CAD	143 (42)
CKD	149 (44)
**Etiology**	
Myocardial infarction	71 (21)
Ischemic heart disease	57 (17)
Dilated cardiomyopathy	45 (13)
Secondary cardiomyopathy	84 (25)
Hypertrophic cardiomyopathy	10 (3)
Valvular heart disease	42 (12)
Pulmonary hypertension	12 (3)
Other causes	20 (6)
**Medication**	
Calcium channel blocker	72 (21)
Beta blocker	236 (69)
ACEi/ARB	253 (74)
Diuretics	170 (50)
Mineralocorticoid blocker	113 (33)
Vitamin K antagonist	42 (12)
Direct oral anticoagulant	42 (12)
Echo image quality (good/fair/poor) (LC)	68/160/113
Echo image quality (good/fair/poor) (RC)	45/144/152

*Data are expressed as numbers (percentages). ACEi/ARB, angiotensin converting enzyme inhibitor/angiotensin receptor blocker; AFib, atrial fibrillation; CAD, coronary artery disease; CKD, chronic kidney disease; DM, diabetes mellitus; HL, hyperlipidemia; HT, hypertension; LC, left chamber; RC, right chamber. Secondary cardiomyopathy includes cardiac sarcoidosis in 28, hypertensive cardiomyopathy in 16, cardiac amyloidosis in 14, tachycardia cardiomyopathy in 4, arrhythmogenic right ventricular cardiomyopathy in 2, cancer therapy-related cardiac dysfunction in 6, alcoholic cardiomyopathy in 4, ampulla cardiomyopathy in 2, peripartum cardiomyopathy in 2, myocarditis in 2, Loeffler endocarditis in 2, eosinophilic myocarditis in 1, hypothyroidism in 1. Valvular heart disease includes mitral valve regurgitation in 9, mitral valve stenosis in 3, aortic valve regurgitation in 9, aortic valve stenosis in 16, pulmonary valve stenosis in 1, post prosthetic valve replacement in 2, infective endocarditis in 2. Other causes include atrial fibrillation in 3, aortic dissection in 2, abdominal aortic aneurysm in 1, pulmonary thromboembolism in 2, Brugada syndrome in 1, torsade de pointes in 1, cardiac tumor in 4, pericarditis in 2, constrictive pericarditis in 1, patent ductus arteriosus in 1, atrial septal defect in 1, left ventricular aneurysm in 1*.

### Echocardiographic Parameters

For 3DE LV parameters, median values of LVEDV index, LVESV index, LVEF, and LVGLS were 90 mL/m^2^ (IQR: 71–124 mL/m^2^), 52 mL/m^2^ (36–85 mL/m^2^), 41% (28–50%), and 12.2% (7.8–15.5 %), respectively. For 3DE RV parameters, the median values of RVEDV index, RVESV index, RVEF by TomTec software were 61 mL/m^2^ (51–76 mL/m^2^), 32 mL/m^2^ (25–42 mL/m^2^), 48% (40–54%), respectively. The median values of RVEDV index, RVESV index, RVEF, RVGCS, RVGLS, and RVGAS by ReVISION software were 61 mL/m^2^ (51–76 mL/m^2^), 33 mL/m^2^ (25–42 mL/m^2^), 47% (39–54%), 19.5% (15.7–23.3%), 15.2% (12.1–18.4%), and 30.0% (24.2–35.4%), respectively. 3D LVEF had a significant correlation with 3D LVGLS (*r* = 0.92) and 3D RVEF (*r* = 0.64). 3D RVEF also had a significant strong correlation with 3D RVGCS (*r* = 0.90), 3D RVGLS (*r* = 0.87), and 3D RVGAS (*r* = 0.93). Echocardiographic parameters are presented in Table 2.

**Table 2:** Clinical and echocardiography parameters in patients with and without a composite of cardiac events (cardiac death, sustained ventricular arrhythmia, or HF hospitalization).

	**Overall (*n* = 341)**	**CE (+) (*n* = 49)**	**CE (-) (*n* = 292)**	***P*-value**	**NNT**
Age (year)	68 [58, 76]	74 [64, 80]	67 [57, 75]	0.005	4.1
Sex (male)	226 (66%)	26 (53%)	200 (68%)	0.034	6.5
BSA (/m^2^)	1.62 [1.50, 1.75]	1.55 [1.44, 1.74]	1.63 [1.51, 1.75]	0.061	5.1
HT	191 (56%)	29 (59%)	162 (55%)	0.6	27.0
DM	101 (30%)	19 (39%)	82 (28%)	0.13	9.4
HL	149 (44%)	22 (45%)	127 (43%)	0.9	71.2
CAD	143 (42%)	19 (39%)	124 (42%)	0.6	27.1
CKD	149 (44%)	29 (59%)	120 (41%)	0.018	5.5
HR (beat/minute)	67 (59, 76)	69 [60, 82]	66 [59, 75]	0.2	8.4
SBP (mmHg)	127 [112, 145]	115 [107, 130]	129 [114, 146]	0.001	3.8
DBP (mmHg)	71 [63, 79]	67 [58, 74]	72 [64, 80]	<0.001	3.1
3D LVEDVI (mL/m^2^)	90 [71, 124]	105 [90, 131]	87 [69, 123]	0.005	5.2
3D LVESVI (mL/m^2^)	52 [36, 85]	68 [52, 102]	49 [33, 83]	<0.001	3.9
3D LVEF (%)	41 [28, 50]	31 [24, 43]	43 [31, 51]	<0.001	2.8
3D LVGLS (%)	12.2 [7.8, 15.5]	8.6 [5.9, 12.3]	12.6 [8.7, 15.9]	<0.001	2.9
3D LAVI max (mL/m^2^)	48 [35, 66]	64 [52, 77]	45 [33, 61]	<0.001	2.7
3D LAVI min (mL/m^2^)	31 [20, 47]	49 [34, 57]	27 [19, 41]	<0.001	2.5
E (cm/sec)	66 [49, 85]	78 [63, 93]	64 [49, 82]	0.007	5.2
A (cm/sec)	70 [51, 90]	79 [41, 96]	69 [52, 89]	0.9	26.7
Average mitral E/e'	11.4 [8.4, 15.2]	13.9 [10.5, 19.1]	10.9 [8.2, 14.6]	<0.001	2.7
SPAP (mmHg)	31 [25, 38]	37 [31, 43]	31 [25, 37]	0.004	3.9
TAPSE (mm)	16.7 [13, 20.6]	14 [11, 18.8]	17 (13, 21)	0.001	3.4
RV s' velocity (cm/sec)	10.6 [8.8, 12.3]	9.6 [8.5, 11.4]	10.7 [8.8, 12.4]	0.074	6.6
**TomTec**					
3D RVEDVI (mL/m^2^)	61 [51, 76]	75 [58, 89]	60 [50, 73]	<0.001	3.9
3D RVESVI (mL/m^2^)	32 [25, 42]	44 [34, 57]	30 [23, 40]	<0.001	2.6
3D RVEF (%)	48 [40, 54]	40 [31, 48]	49 [41, 55]	<0.001	2.1
**ReVISION**					
3D RVEDVI (mL/m^2^)	61 [51, 76]	75 [58, 89]	61 [50, 73]	<0.001	3.9
3D RVESVI (mL/m^2^)	33 [25, 42]	44 [34, 57]	31 [24, 41]	<0.001	2.7
3D RVEF (%)	47 [39, 54]	39 [32, 46]	48 [41, 54]	<0.001	2.1
3D RVGCS (%)	19.5 [15.7, 23.3]	15.9 [12.1, 20.3]	20.0 [16.5, 23.7]	<0.001	2.4
3D RVGLS (%)	15.2 [12.1, 18.4]	12.4 [9.7, 15.4]	15.7 [12.8, 18.8]	<0.001	2.5
3D RVGAS (%)	30.0 [24.2, 35.4]	23.3 [17.8, 30.2]	30.5 [25.4, 36.0]	<0.001	2.2

*Data are expressed as numbers (percentages) or medians [interquartile ranges]. 3D, three-dimensional; BSA, body surface area; CE, a composite of cardiac event (cardiac death, sustained ventricular arrhythmia, or heart failure hospitalization); DBP, diastolic blood pressure; HF, heat failure; HR, heart rate; LAVI max (min), left atrial maximum (minimum) volume index; LVED(S)VI, left ventricular end-diastolic (systolic) volume index, LVEF, left ventricular ejection fraction; LVGLS, left ventricular global longitudinal strain; NNT, number needed to treat; RV, right ventricular; RVED(S)VI, right ventricular end-diastolic (systolic) volume index; RVEF, right ventricular ejection fraction; RVGAS, right ventricular global area strain; RVGCS, right ventricular global circumferential strain; RVGLS, right ventricular global longitudinal strain; SBP, systolic blood pressure; SPAP, systolic pulmonary arterial pressure; TAPSE, tricuspid annular plane systolic excursion. Other abbreviations are the same as in Table 1*.

### Association With “Cardiac Death, Ventricular Tachyarrhythmia, or HF Hospitalization”

**Figure 3:**
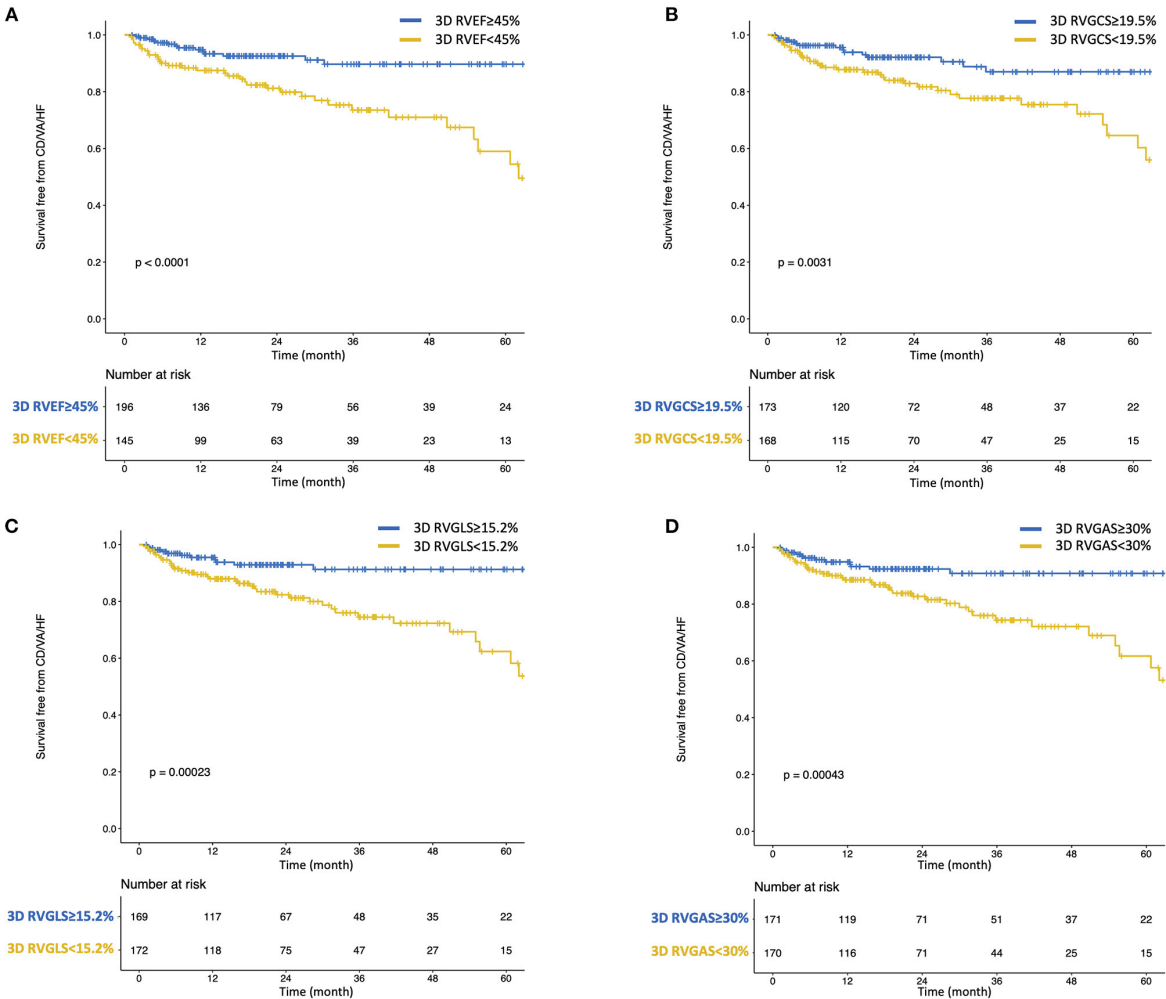
Kaplan-Meier survival curves for cardiac death, ventricular tachyarrhythmia, or heart failure hospitalization stratified by predefined cut-off value of 3D RVEF **(A)** and median values of 3D RVGCS **(B)**, 3D RVGLS **(C)**, and 3D RVGAS **(D)**. CD, cardiac death; HF, heart failure; VA, ventricular tachyarrhythmia.

**Figure 4:**
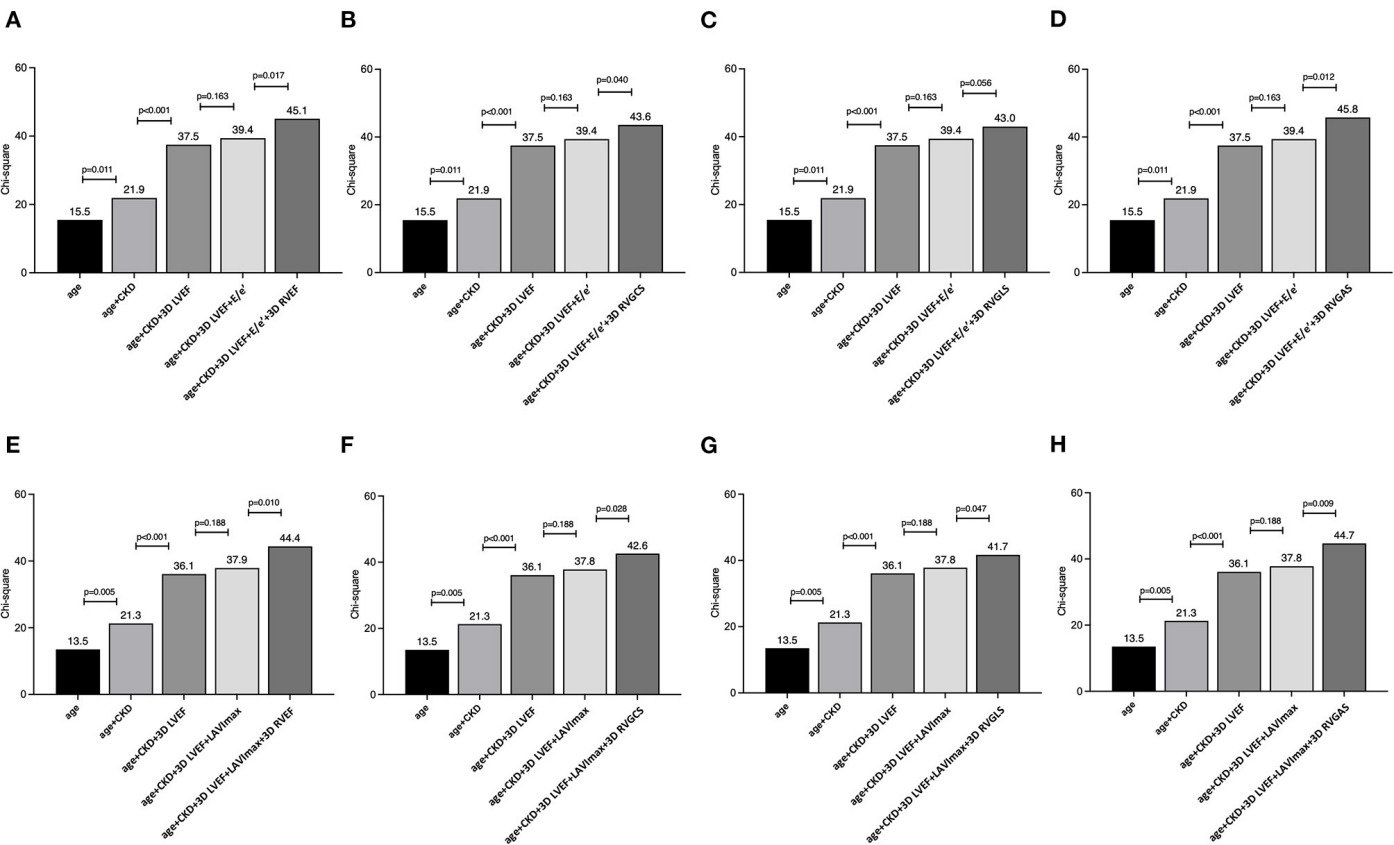
The nested regression model to evaluate the incremental value of 3D RVEF, 3D RVGCS, 3D RVGLS, and 3D RVGAS for cardiac death, ventricular tachyarrhythmia, or HF hospitalization. χ^2^ scores show that 3D RVEF, 3D RVGCS, and 3D RVGAS have significant incremental value for prediction of a composite of cardiac events in addition to models, including age, chronic kidney disease, left ventricular ejection fraction, and average mitral E/e' **(A–D)**. χ^2^ scores show that 3D RVEF, all 3D global strains have significant incremental value for prediction over the models, including age, chronic kidney disease, left ventricular ejection fraction, and maximum 3D left atrial volume index **(E–H)**.

**Figure 5:**
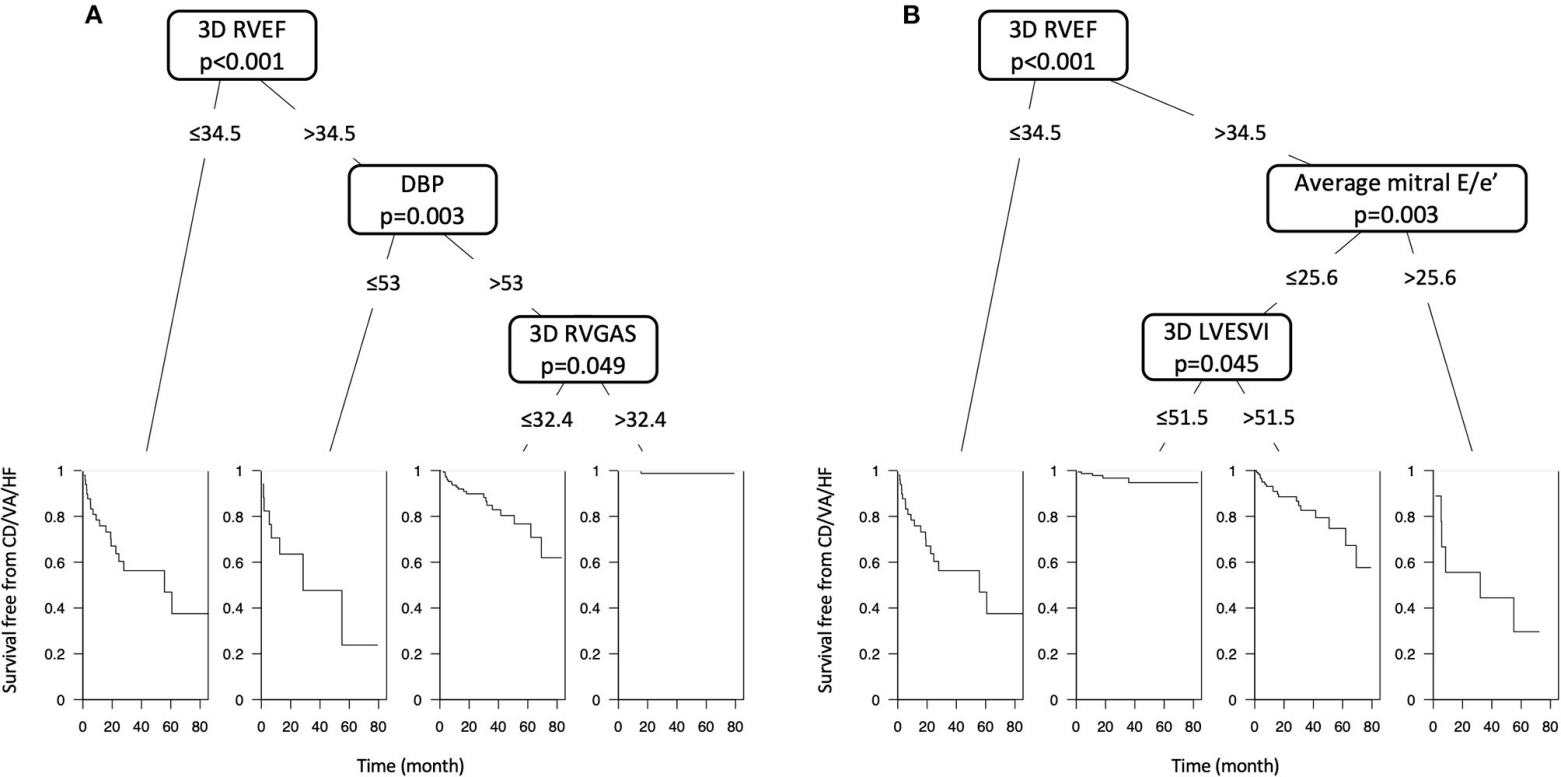
**(A)** Classification and regression tree (CART) analysis, including twenty-two clinical and echocardiographic parameters. CART selected 3D RVEF (cut-off value: 34.5%) first, followed by DBP (cut-off value: 53 mmHg) and 3D RVGAS (cut-off value: 32.4%), resulting in classification into two high-risk groups (*n* = 67), one intermediate-risk group (*n* = 154), and one low-risk group (*n* = 120). **(B)** CART analysis, including fifteen echocardiographic parameters. CART selected 3D RVEF (cut-off value: 34.5%) first, followed by average mitral E/e' (cut-off value: 25.6) and 3D LVESVI (cut-off value: 51.5 mL/m^2^), resulting in classification into two high-risk groups (*n* = 59), one intermediate-risk group (*n* = 126), and one low-risk group (*n* = 156).

During a median of 19.8 (IQR: 9.0–38.5) months of follow-up, 49 patients reached a composite of cardiac events, 14 of whom suffered cardiac death. Thirty patients were HF hospitalization, and 5 patients developed sustained ventricular tachyarrhythmia. Of the 14 patients with cardiac death, worsened heart failure was the cause of death in eight patients, ventricular tachyarrhythmia was the cause of death in three, sudden cardiac death was the cause in two, and myocardial infarction was the cause of death in one patient. Table 2 presents clinical and echocardiographic parameters between patients with and without cardiac events, and their NNTs. NNT was smallest in 3D RVEF (2.1), followed by 3D RVGAS (2.2), 3D RVGCS (2.4), 3D RVGLS (2.5), 3D minimum LA volume index (2.5), and 3D RVESV index (2.7). Kaplan-Meier survival analysis of 3D RVEF which were divided into binary groups using predefined cut-off value of 45% and that of 3D RVGCS, 3D RVGLS, and 3D RVGAS, which were divided into binary groups using median values showed that all four parameters had significant discriminatory power for “cardiac death, ventricular tachyarrhythmia, or HF hospitalization” (Figure 3). In univariate Cox proportional hazard analysis, 3D RVEF (HR: 0.93, 95%CI: 0.91–0.96), 3D RVGCS (HR: 0.88, 95%CI: 0.83–0.93), 3D RVGLS (HR: 0.85, 95%CI: 0.79–0.91), and 3D RVGAS (HR: 0.91, 95%CI: 0.88–0.94) were significantly associated with “cardiac death, ventricular tachyarrhythmia, or HF hospitalization” (Supplementary Table 1). The corresponding values of C-Statistic were 0.71 (95%CI: 0.63–0.78), 0.68 (0.60–0.76), 0.68 (0.60–0.76), and 0.70 (0.62–0.77), respectively. There were no statistically significant differences in C-Statics between 3D RVEF and 3D RV strain parameters. We also performed a dichotomous univariate analysis using the cutoff values based on previous reports (3, 13) (Table 3). 3D RVGLS < 15% had a similar hazard ratio compared with 3D RVEF < 45%. Multivariate Cox proportional hazard analysis showed that 3D RVEF and 3D RV strains were associated with future cardiac events, after adjusting age, chronic kidney disease (CKD), 3D LVEF, and average mitral E/e', 3D maximal left atrial volume index (LAVI), or TAPSE (Supplementary Table 2). Incremental values of 3D RVEF and three RV 3D strains are shown in Figure 4. When 3D RVEF, 3D RVGCS, or 3D RVGAS were added to the model including age, CKD, 3D LVEF and average mitral E/e', chi-squared values increased significantly. When either of four parameters were added to the model including age, CKD, 3D LVEF and 3D maximal LAVI, the chi-square value increased significantly. Figure 5 shows results of CART analysis. When 22 clinical and echocardiographic parameters were used, including age, gender, body surface area, CKD, heart rate, systolic blood pressure, diastolic blood pressure (DBP), 3D LVEDVI, 3D LVESVI, 3D LVEF, 3D LVGLS, 3D maximal LAVI, 3D minimal LAVI, E/e', TAPSE, RV s', 3D RVEDVI, 3D RVESVI, 3D RVEF, 3D RVGCS, 3D RVGLS, and 3D RVGAS. CART selected 3D RVEF (cut-off value: 34.5%) first, followed by DBP at the time of echocardiography examination (cut-off value: 53 mmHg) and 3D RVGAS (cut-off value: 32.4%), resulting in classification into two high-risk groups, one intermediate-risk group, and one low-risk group (Figure 5A). If we included 15 echocardiography parameters, CART selected 3D RVEF (cut-off value: 34.5%) first, followed by average mitral E/e' (cut-off value: 25.6) and 3D LVESVI (cut-off value: 51.5 mL/m^2^), resulting in classification into two high-risk groups, one intermediate-risk group, and one low-risk group (Figure 5B).

**Table 3:** Univariate cox proportional hazards analysis with dichotomous variables for “cardiac death, sustained ventricular arrhythmia, or HF hospitalization.”

**Variables**	**Hazard ratio**	**95% CI**	***P*-value**
3D LVEF < 50 %	5.66	1.75–18.3	0.004
3D LVEF < 40 %	2.71	1.47–4.99	0.001
3D LVEF < 30 %	2.11	1.20–3.73	0.010
3D LVGLS < 16 %	2.92	1.15–7.37	0.024
3D LVGLS < 13 %	3.05	1.55–6.00	0.001
3D LVGLS < 10 %	3.48	1.91–6.34	<0.001
3D LAVI max > 34 mL/m^2^	4.82	1.91–12.2	<0.001
Average mitral E/e' > 14	1.78	1.01–3.15	0.046
TR > 2.8 m/s	2.60	1.36–4.98	0.004
TAPSE < 17 mm	2.15	1.18–3.90	0.012
TAPSE < 13 mm	2.62	1.48–4.64	<0.001
TAPSE < 10 mm	2.38	1.11–5.09	0.025
RV s' < 9.5 cm/sec	1.98	1.05–3.72	0.034
RV s' < 7.5 cm/sec	1.07	0.42–2.73	0.9
RV s' < 5 cm/sec	2.26	0.31–16.5	0.4
3D RVEF < 45 %	3.22	1.75–5.92	<0.001
3D RVEF < 40 %	3.09	1.76–5.42	<0.001
3D RVEF < 35 %	3.91	2.20–6.95	<0.001
3D RVEF < 30 %	3.81	1.94–7.47	<0.001
3D RVGCS < 19 %	2.70	1.49–4.92	<0.001
3D RVGLS < 15 %	3.22	1.70–6.08	<0.001
3D RVGAS < 30 %	2.97	1.57–5.61	<0.001

*CI, confidence interval; TR, tricuspid regurgitation velocity. Other abbreviations are the same as in Table 2*.

### Association With HF Hospitalization

**Figure 6:**
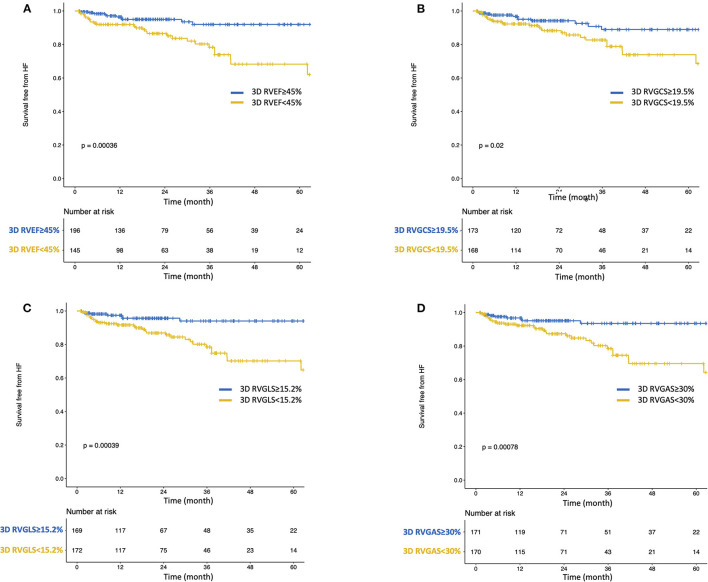
Kaplan-Meier survival curves for heart failure hospitalization stratified by predefined cut-off value of 3D RVEF **(A)** and median values of 3D RVGCS **(B)**, 3D RVGLS **(C)**, and 3D RVGAS **(D)**.

During a median of 19.7 (IQR: 8.7–37.7) months of follow-up, 37 patients reached HF hospitalization. Supplementary Table 3 presents clinical and echocardiographic parameters between patients with and without HF hospitalization, and their NNTs. Figure 6 showed Kaplan-Meier survival analysis of 3D RVEF, 3D RVGCS, 3D RVGLS, and 3D RVGAS divided into binary groups using the aforementioned cutoff values. In univariate analysis, 3D RVEF (HR: 0.93, 95%CI: 0.90–0.96), 3D RVGCS (HR: 0.88, 95%CI: 0.83–0.94), 3D RVGLS (HR: 0.84, 95%CI: 0.78–0.91), 3D RVGAS (HR: 0.91, 95%CI: 0.87–0.95) were significantly associated with HF hospitalization (Supplementary Table 4). Table 4 shows a dichotomous univariate analysis for several echocardiographic parameters using the cutoff values based on previous reports (3, 13). 3D RVGLS < 15% had a similar hazard ratio for HF hospitalization compared with 3D RVEF < 45%.

**Table 4:** Univariate cox proportional hazards analysis with dichotomous variables for HF hospitalization.

**Variables**	**Hazard ratio**	**95% CI**	***P*-value**
3D LVEF < 50 %	4.15	1.27–13.6	0.019
3D LVEF < 40 %	2.85	1.40–5.80	0.004
3D LVEF < 30 %	2.02	1.04–3.89	0.037
3D LVGLS < 16 %	3.96	1.18–12.6	0.025
3D LVGLS < 13 %	3.82	1.67–8.75	0.001
3D LVGLS < 10 %	4.01	1.97–8.15	<0.001
3D LAVI max > 34 mL/m^2^	6.08	1.86–19.8	0.003
Average mitral E/e' > 14	1.73	0.90–3.34	0.10
TR > 2.8 m/s	3.19	1.51–6.77	0.002
TAPSE < 17 mm	2.16	1.08–4.30	0.028
TAPSE < 13 mm	2.38	1.22–4.63	0.011
TAPSE < 10 mm	1.94	0.75–4.99	0.2
RV s' < 9.5 cm/sec	1.68	0.82–3.33	0.2
RV s' < 7.5 cm/sec	0.79	0.24–2.59	0.7
RV s' < 5 cm/sec	0.00	0.00–Inf	0.9
3D RVEF < 45 %	3.36	1.66–6.82	<0.001
3D RVEF < 40 %	2.85	1.49–5.43	0.001
3D RVEF < 35 %	3.50	1.78–6.89	<0.001
3D RVEF < 30 %	4.32	2.03–9.22	<0.001
3D RVGCS < 19 %	2.40	1.22–4.73	0.011
3D RVGLS < 15 %	3.61	1.70–7.67	<0.001
3D RVGAS < 30 %	3.37	1.59–7.16	0.002

*Abbreviations are the same as in Tables 2, 3*.

### Reliability of the ReVISION Method

To evaluate the reliability of the ReVISION method, 3D RVEF values by the ReVISION method were compared to corresponding values obtained from TomTec software or CMR. The prognostic value was also evaluated among three RVEFs. A correlation coefficient of RVEF between the ReVISION method and TomTec software was 0.99 and that between the ReVISION method and CMR was 0.67 (Supplementary Figure 1). The prognostic values of primary and secondary endpoints were comparable among RVEFs assessed by the three methods (Table 5).

**Table 5:** Comparison of the prognostic value of RVEF among ReVISION method and the other methods.

**Variables**	**Hazard ratio**	**95% CI**	***P*-value**
**“Cardiac death, sustained ventricular arrhythmia, or HF hospitalization”**			
3D RVEF by ReVISION method	0.93	0.91–0.96	<0.001
3D RVEF by TomTec software	0.93	0.91–0.96	<0.001
RVEF by CMR	0.96	0.94–0.99	0.002
**HF hospitalization**			
3D RVEF by ReVISION method	0.93	0.90–0.96	<0.001
3D RVEF by TomTec software	0.93	0.90–0.96	<0.001
RVEF by CMR	0.96	0.93–0.98	0.002

*CMR, cardiac magnetic resonance. TomTec software was 4D RV function 3. Other abbreviations are the same as in Tables 2, 3*.

### Reproducibility

The intra- and inter-observer variability of 3D RVEDV, 3D RVESV and 3D RVEF was 4.5–5.4% [intraclass correlation coefficients (ICC): 0.92–0.97] and 7.4–9.2% (ICC: 0.86–0.93), respectively (Supplementary Table 5).

## Discussion

To the best of our knowledge, this is the first report of the prognostic utility of RV 3D strains in patients with diverse cardiac diseases. The major findings of our study can be summarized as follows:, (i) Univariate analysis showed that 3D RVEF and RV 3D strains were associated with future outcomes, (ii) Multivariate analysis revealed that 3D RVEF, 3D RVGCS and 3D RVGAS are independently associated with cardiac events after adjusting for age, CKD, 3D LVEF, and average mitral E/e'. 3D RVEF and all 3D RV global strains are independently associated with cardiac events after adjusting for age, CKD, 3D LVEF, and 3D maximum LAVI, (iii) CART selected 3D RVEF first, followed by DBP and 3D RVGAS, which divided the patients into four groups stratified for risk of cardiac events of different degrees.

### Previous Studies

Echocardiographic cardiac function analysis has focused mainly on left cardiac chambers, including LVEF, LVGLS, and left atrial volumes and function. The right ventricle, on the other hand, has been regarded for many years as the neglected or forgotten chamber of the heart, with less relevance to RV disease as a primary cardiac disease (14). However, the importance of RV function, especially RVEF, has been recently demonstrated in management and prognostication of cardiac disease (4–6). It has also been reported that patients who had preserved LVEF and reduced RVEF had significantly worse prognoses than patients with reduced LVEF, but preserved RVEF (4, 15), and more attention is now being paid to the right heart chambers.

RV pump function consists of three main mechanisms: (i) shortening of the longitudinal axis with traction of the tricuspid annulus toward the apex; (ii) inward (radial) movement of the RV free wall; (iii) bulging of the interventricular septum into the RV during LV contraction and stretching of the free wall over the septum (7, 16). Impairment of these mechanisms may vary depending on cardiac diseases or conditions. Hence, Lakatos et al. (8) developed the ReVISION method, a 3DE-based solution for quantification of the relative contributions of longitudinal, radial, and antero-posterior shortening to global RVEF. Assessments of RVGCS, RVGLS, and RVGAS have also been implemented. Atsumi et al. (17) first demonstrated the reliability and clinical feasibility of RV 3D strains in animal studies. Ishizu et al. (18) showed that RV 3D strains are associated with impaired RV function in adult patients with a wide array of cardiovascular problems. In these studies, a 3D wall motion tracking algorithm for the RV was used. We reported normal values of RV 3D strains in healthy volunteers using the ReVISION software (8). Recently, RV 3D strains were shown to be associated with short-term outcomes in patients undergoing cardiac surgery (19). However, no studies have investigated whether RV 3D strains provide prognostic information in patients with cardiovascular disease.

### Current Study

Univariate and multivariate Cox proportional hazard analysis revealed that not only 3D RVEF but also 3D RV global strains were significantly associated with a composite of cardiac events as well as HF hospitalization. However, because of the collinearity of 3D RVEF and RV 3D strains, multivariate analyses including both 3D RVEF and RV 3D strains were not conducted in this study.

In the CART analysis, 3D RVEF was selected first, followed by DBP, and finally 3D RVGAS, when we included clinical and echocardiography parameters. Selection of 3D RVGAS makes sense because of its smaller NNT next to that of 3D RVEF and because its value reflects strain values in both longitudinal and circumferential directions. Previous publications have reported that lower DBPs are associated with worse prognoses (20–22). In addition, low systolic and mean blood pressures are associated with increased mortality in heart failure patients (22, 23). When we included 15 echocardiography parameters, CART selected 3D RVEF first, followed by LV diastolic function parameter (mitral E/e') and LV systolic function parameter (3D LVESVI). The results suggest that among echocardiographic parameters, 3D RVEF is more important than LV diastolic and systolic parameters to predict future outcome, which agreed with the previous study in patients with asymptomatic aortic stenosis (24).

In the present study, RV 3D strain had prognostic value equivalent to that of 3D RVEF, but not better than 3D RVEF. This may be due to the inclusion of left-sided heart disease of various etiologies and the inclusion of patients with preserved or impaired LV function, which may have resulted in different mechanisms of RV dysfunction among patients. Left-sided heart disease was also thought to diffusely impair the right ventricle. As a result, 3D RV strain, which is an indicator of one or two directional motion components, was equivalent but not superior to 3D RVEF, which is a global indicator. This was also consistent with the fact that for RV 3D strain values, 3D RVGAS, a multidimensional index, was a better parameter for prognosis than 3D RVGCS or 3D RVGLS. Combining these parameters with RVEF may allow more detailed stratification of patient prognosis, especially in a specific type of cardiovascular diseases.

The cut-off values of 3D RVGCS, 3D RVGLS, and 3D RVGAS found for outcome analysis in this study were 19.5, 15.2, and 30.0%, respectively. These values were correctly below the lower limit of normality (LLN) of 3D RVGCS (LLN: 21.3%), 3D RVGLS (LLN: 24.7%), and 3D RVGAS (LLN: 34.8%) reported by Lakatos et al. (8), which is in agreement with the prognostic cut-off value for LVGLS that is also below the LLN for LVGLS (i.e., <16%) (25–27), since cut-off values to estimate worse outcomes should be below the LLN of the parameter analyzed. 3D RVGLS <15% had a similar hazard ratio compared with 3D RVEF < 45% for the association of a composite of cardiac events as well as HF hospitalization. Further study should be required to validate whether 3D RVGLS of 15% is an optimal cut-off value for prognostication. It is also important to determine whether 3D RVGLS provides useful prognostic information in patients whose 3D RVEF is preserved.

### Study Limitations

Some limitations must be acknowledged. This study was single-center, retrospective, observational study that included patients selected from the CMR database. Selection bias should be recognized because only patients undergoing CMR were included, which might be biased toward certain left-sided cardiac conditions. Some echocardiographic parameters and information on the severity of valvular heart disease were not used for the analysis. ReVISION software relies on 3D RV endocardial meshes generated using vendor-independent, commercially available 3DE speckle tracking software. The relatively small number of events in this study did not allow for extensive subgroup analysis. Further studies are needed to investigate the potential usefulness of RV 3D strains in specific cardiac diseases. The decision tree obtained from the CART analysis has been optimized for our population and needs to be externally validated in future studies.

### Conclusions

RV 3D strains provided equivalesnt prognostic usefulness compared with 3D RVEF in patients with diverse cardiac diseases. Combining these parameters with 3D RVEF may allow for a more detailed stratification of patient prognoses.

## Data Availability Statement

The raw data supporting the conclusions of this article will be made available by the authors, without undue reservation.

## Ethics Statement

The studies involving human participants were reviewed and approved by the Ethics Committee at the University of Occupational and Environmental Health. Written informed consent for participation was not required for this study in accordance with the national legislation and the institutional requirements.

## Author Contributions

TK: conceptualization, data curation, data analysis, investigation, methodology, and writing-original draft. AK: investigation, methodology, and writing-original draft. YN: data curation. MT, AF, and BL: investigation and methodology. MT: conceptualization, data analysis, investigation, methodology, writing-original draft, and supervision. All authors contributed to the article and approved the submitted version.

## Conflict of Interest

The authors declare that the research was conducted in the absence of any commercial or financial relationships that could be construed as a potential conflict of interest.

## Publisher's Note

All claims expressed in this article are solely those of the authors and do not necessarily represent those of their affiliated organizations, or those of the publisher, the editors and the reviewers. Any product that may be evaluated in this article, or claim that may be made by its manufacturer, is not guaranteed or endorsed by the publisher.

## Supplementary Material

Supplementary Figure 1 Correlation plot between RVEF by ReVISION method and 4D RV Function 3 or CMR.

Supplementary Table 1 Univariate Cox proportional hazard analysis for “cardiac death, ventricular tachyarrythmia, or HF hospitalization.” 3D, three-dimensional; BSA, body surface area; CAD, coronary artery disease; CI, confidence interval; CKD, chronic kidney disease; DBP, diastolic blood pressure; DM, diabetes mellitus; HF, heart failure; HL, hyperlipidemia; HR, heart rate; HT, hypertension; LAVI, left atrial volume index; LVED(S)VI, left ventricular end-diastolic (systolic) volume index, LVEF, left ventricular ejection fraction; LVGLS, left ventricular global longitudinal strain; RV, right ventricular; RVED(S)VI, right ventricular end-diastolic (systolic) volume index; RVEF, right ventricular ejection fraction; RVGAS, right ventricular global area strain; RVGCS, right ventricular global circumferential strain; RVGLS, right ventricular global longitudinal strain; SBP, systolic blood pressure; SPAP, systolic pulmonary arterial pressure; TAPSE, tricuspid annular plane systolic excursion.

Supplementary Table 2 Multivariable Cox proportional hazard analysis for “cardiac death, ventricular tachyarrhythmia, or HF hospitalization.” HR, hazard ratio. Other abbreviations are the same as in Supplementary Table 1.

Supplementary Table 3 Clinical and echocardiography parameters in patients with and without HF hospitalization. Data are expressed as numbers (percentages) or medians [interquartile ranges]. NNT, number needed to treat. Other abbreviations are the same as in Supplementary Table 1.

Supplementary Table 4 Univariate Cox proportional hazard analysis for HF hospitalization. Abbreviations are the same as in Supplementary Table 1.

Supplementary Table 5 Result of reproducibility analysis for right ventricular volumes and right ventricular ejection fraction. ICC, intraclass correlation coefficients; IQR, interquartile ranges. Other abbreviations are the same as in Supplementary Table 1.
