# Supplementary figures and images for: Prognostic Value of Right Ventricular Strains Using Novel Three-Dimensional Analytical Software in Patients With Cardiac Disease
Source: Front Cardiovasc Med. 2022 Feb 25;9:837584. doi: 10.3389/fcvm.2022.837584 (PMC8914046; doi:10.3389/fcvm.2022.837584)

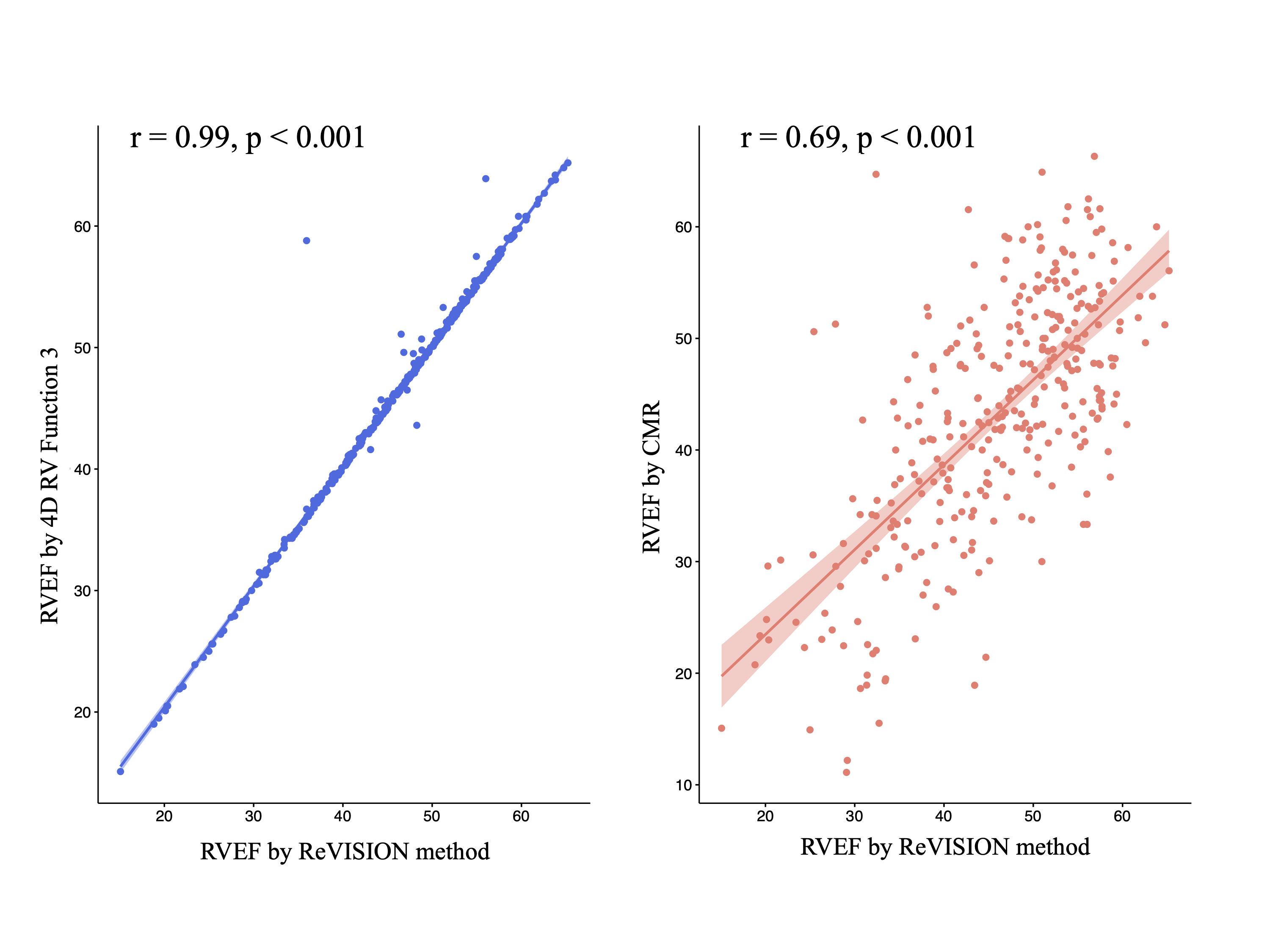

Supplement: Supplementary Figure 1 — Correlation plot between RVEF by ReVISION method and 4D RV Function 3 or CMR. [file Image_1.TIFF]
