# Supplementary material for: Prognostic Value of Right Ventricular Strains Using Novel Three-Dimensional Analytical Software in Patients With Cardiac Disease
Source: Front Cardiovasc Med. 2022 Feb 25;9:837584. doi: 10.3389/fcvm.2022.837584 (PMC8914046; doi:10.3389/fcvm.2022.837584)
Supplement: Supplementary Table 1 — Univariate Cox proportional hazard analysis for “cardiac death, ventricular tachyarrythmia, or HF hospitalization.” 3D, three-dimensional; BSA, body surface area; CAD, coronary artery disease; CI, confidence interval; CKD, chronic kidney disease; DBP, diastolic blood pressure; DM, diabetes mellitus; HF, heart failure; HL, hyperlipidemia; HR, heart rate; HT, hypertension; LAVI, left atrial volume index; LVED(S)VI, left ventricular end-diastolic (systolic) volume index, LVEF, left ventricular ejection fraction; LVGLS, left ventricular global longitudinal strain; RV, right ventricular; RVED(S)VI, right ventricular end-diastolic (systolic) volume index; RVEF, right ventricular ejection fraction; RVGAS, right ventricular global area strain; RVGCS, right ventricular global circumferential strain; RVGLS, right ventricular global longitudinal strain; SBP, systolic blood pressure; SPAP, systolic pulmonary arterial pressure; TAPSE, tricuspid annular plane systolic excursion. [file Table_1.docx]

**Supplementary Table 1: Univariate Cox proportional hazard analysis for “cardiac death, ventricular tachyarrythmia, or HF hospitalization”**

| Variables | Hazard ratio | 95% CI | P-value |
| --- | --- | --- | --- |
| Age (year) | 1.04 | 1.02 to 1.07 | 0.002 |
| Gender (male) | 0.66 | 0.38 to 1.17 | 0.2 |
| BSA (/m^2^) | 0.31 | 0.08 to 1.25 | 0.10 |
| HR (beat/min) | 1.02 | 0.99 to 1.04 | 0.2 |
| SBP (mmHg) | 0.98 | 0.97 to 1.00 | 0.016 |
| DBP (mmHg) | 0.95 | 0.93 to 0.98 | <0.001 |
| HT (%) | 1.20 | 0.68 to 2.13 | 0.5 |
| DM (%) | 1.51 | 0.85 to 2.68 | 0.2 |
| HL (%) | 1.02 | 0.58 to 1.79 | 0.9 |
| CAD (%) | 0.93 | 0.53 to 1.66 | 0.8 |
| CKD (%) | 2.73 | 1.53 to 4.89 | <0.001 |
| 3D LVEDVI (mL/m^2^) | 1.01 | 1 to 1.01 | 0.069 |
| 3D LVESVI (mL/m^2^) | 1.01 | 1 to 1.02 | 0.012 |
| 3D LVEF (%) | 0.96 | 0.94 to 0.98 | <0.001 |
| 3D LVGLS (%) | 0.89 | 0.84 to 0.95 | <0.001 |
| 3D LAVI max (mL/m^2^) | 1.02 | 1.01 to 1.03 | <0.001 |
| 3D LAVI min (mL/m^2^) | 1.02 | 1.01 to 1.03 | <0.001 |
| E wave (cm/sec) | 1.01 | 1.00 to 1.01 | 0.2 |
| A wave (cm/sec) | 1.00 | 0.99 to 1.01 | 0.7 |
| Average mitral E/e’ | 1.07 | 1.03 to 1.10 | <0.001 |
| SPAP (mmHg) | 1.03 | 1.01 to 1.05 | 0.005 |
| TAPSE (mm) | 0.90 | 0.85 to 0.96 | <0.001 |
| RV s’ (cm/sec) | 0.90 | 0.80 to 1.01 | 0.081 |
| TomTec |  |  |  |
| 3D RVEDVI (mL/m^2^) | 1.01 | 1 to 1.02 | 0.011 |
| 3D RVESVI (mL/m^2^) | 1.02 | 1.01 to 1.03 | <0.001 |
| 3D RVEF (%) | 0.93 | 0.91 to 0.96 | <0.001 |
| ReVISION |  |  |  |
| 3D RVEDVI (mL/m^2^) | 1.01 | 1.00 to 1.02 | 0.011 |
| 3D RVESVI (mL/m^2^) | 1.02 | 1.01 to 1.03 | <0.001 |
| 3D RVEF (%) | 0.93 | 0.91 to 0.96 | <0.001 |
| 3D RVGCS (%) | 0.88 | 0.83 to 0.93 | <0.001 |
| 3D RVGLS (%) | 0.85 | 0.79 to 0.91 | <0.001 |
| 3D RVGAS (%) | 0.91 | 0.88 to 0.94 | <0.001 |

3D, three-dimensional; BSA, body surface area; CAD, coronary artery disease; CI, confidence interval; CKD, chronic kidney disease; DBP, diastolic blood pressure; DM, diabetes mellitus; HF, heart failure; HL, hyperlipidemia; HR, heart rate; HT, hypertension; LAVI, left atrial volume index; LVED(S)VI, left ventricular end-diastolic (systolic) volume index, LVEF, left ventricular ejection fraction; LVGLS, left ventricular global longitudinal strain; RV, right ventricular; RVED(S)VI, right ventricular end-diastolic (systolic) volume index; RVEF, right ventricular ejection fraction; RVGAS, right ventricular global area strain; RVGCS, right ventricular global circumferential strain; RVGLS, right ventricular global longitudinal strain; SBP, systolic blood pressure; SPAP, systolic pulmonary arterial pressure; TAPSE, tricuspid annular plane systolic excursion.
