# Supplementary material for: Prognostic Value of Right Ventricular Strains Using Novel Three-Dimensional Analytical Software in Patients With Cardiac Disease
Source: Front Cardiovasc Med. 2022 Feb 25;9:837584. doi: 10.3389/fcvm.2022.837584 (PMC8914046; doi:10.3389/fcvm.2022.837584)
Supplement: Supplementary Table 2 — Multivariable Cox proportional hazard analysis for “cardiac death, ventricular tachyarrhythmia, or HF hospitalization.” HR, hazard ratio. Other abbreviations are the same as in Supplementary Table 1. [file Table_2.docx]

**Supplementary Table 2: Multivariable Cox proportional hazard analysis for for “cardiac death, ventricular tachyarrhythmia, or HF hospitalization”**

|  | 3D RVEF model | | 3D RVGCS model | | 3D RVGLS model | | 3D RVGAS model | |
| --- | --- | --- | --- | --- | --- | --- | --- | --- |
|  | HR (95% CI) | p-value | HR (95% CI) | p-value | HR (95% CI) | p-value | HR (95% CI) | p-value |
| Age | 1.06 (1.02 – 1.09) | <0.001 | 1.05 (1.02 – 1.09) | <0.001 | 1.05 (1.02 – 1.09) | 0.001 | 1.05 (1.02 – 1.09) | 0.001 |
| CKD | 1.43 (0.76 – 2.70) | 0.3 | 1.55 (0.83 – 2.91) | 0.2 | 1.50 (0.80 – 2.82) | 0.2 | 1.53 (0.81 – 2.87) | 0.2 |
| Average mitral E/e’ | 1.02 (0.98 – 1.06) | 0.4 | 1.02 (0.98 – 1.06) | 0.3 | 1.02 (0.99 – 1.06) | 0.2 | 1.02 (0.98 – 1.06) | 0.3 |
| 3D LVEF | 0.98 (0.95 – 1.01) | 0.12 | 0.98 (0.95 – 1.01) | 0.11 | 0.98 (0.95 – 1.01) | 0.14 | 0.99 (0.95 – 1.02) | 0.4 |
| 3D RVEF | 0.96 (0.92 – 0.99) | 0.015 |  |  |  |  |  |  |
| 3D RVGCS |  |  | 0.93 (0.86 – 1.00) | 0.038 |  |  |  |  |
| 3D RVGLS |  |  |  |  | 0.91 (0.83 – 1.00) | 0.058 |  |  |
| 3D RVGAS |  |  |  |  |  |  | 0.93 (0.88 – 0.98) | 0.012 |

|  | 3D RVEF model | | 3D RVGCS model | | 3D RVGLS model | | 3D RVGAS model | |
| --- | --- | --- | --- | --- | --- | --- | --- | --- |
|  | HR (95% CI) | p-value | HR (95% CI) | p-value | HR (95% CI) | p-value | HR (95% CI) | p-value |
| Age | 1.05 (1.02 – 1.08) | 0.001 | 1.05 (1.02 – 1.08) | 0.001 | 1.05 (1.02 – 1.08) | 0.003 | 1.05 (1.02 – 1.08) | 0.002 |
| CKD | 1.53 (0.81 – 2.88) | 0.2 | 1.68 (0.89 – 3.15) | 0.11 | 1.61 (0.86 – 3.02) | 0.14 | 1.65 (0.88 – 3.09) | 0.12 |
| 3D LAVI max | 1.00 (0.99 –1.01) | 0.6 | 1.00 (0.99 – 1.02) | 0.5 | 1.01 (1.00 – 1.02) | 0.3 | 1.02 (0.99 – 1.01) | 0.5 |
| 3D LVEF | 0.98 (0.95 – 1.01) | 0.2 | 0.98 (0.95 – 1.01) | 0.2 | 0.98 (0.95 – 1.01) | 0.2 | 0.99 (0.96 – 1.02) | 0.4 |
| 3D RVEF | 0.95 (0.92 – 0.99) | 0.010 |  |  |  |  |  |  |
| 3D RVGCS |  |  | 0.92 (0.86 – 0.99) | 0.027 |  |  |  |  |
| 3D RVGLS |  |  |  |  | 0.91 (0.83 – 1.00) | 0.050 |  |  |
| 3D RVGAS |  |  |  |  |  |  | 0.93 (0.88 – 0.98) | 0.009 |

|  | 3D RVEF model | | 3D RVGCS model | | 3D RVGLS model | | 3D RVGAS model | |
| --- | --- | --- | --- | --- | --- | --- | --- | --- |
|  | HR (95% CI) | p-value | HR (95% CI) | p-value | HR (95% CI) | P-value | HR (95% CI) | p-value |
| Age | 1.06 (1.02 – 1.09) | <0.001 | 1.05 (1.02 – 1.09) | <0.001 | 1.05 (1.02 – 1.08) | 0.001 | 1.05 (1.02 – 1.08) | <0.001 |
| CKD | 1.50 (0.80 – 2.81) | 0.2 | 1.71 (0.91 – 3.20) | 0.091 | 1.65 (0.88 – 3.09) | 0.12 | 1.70 (0.91 – 3.18) | 0.095 |
| TAPSE | 1.06 (0.97 – 1.16) | 0.2 | 1.03 (0.95 – 1.12) | 0.486 | 1.03 (0.94 – 1.13) | 0.5 | 1.07 (0.98 – 1.17) | 0.11 |
| 3D LVEF | 0.98 (0.95 – 1.01) | 0.2 | 0.98 (0.95 – 1.01) | 0.134 | 0.98 (0.95 – 1.01) | 0.2 | 0.99 (0.96 – 1.03) | 0.7 |
| 3D RVEF | 0.93 (0.88 – 0.98) | 0.004 |  |  |  |  |  |  |
| 3D RVGCS |  |  | 0.89 (0.81 – 0.98) | 0.019 |  |  |  |  |
| 3D RVGLS |  |  |  |  | 0.87 (0.76 – 1.00) | 0.054 |  |  |
| 3D RVGAS |  |  |  |  |  |  | 0.88 (0.82 – 0.95) | 0.002 |

HR, hazard ratio. Other abbreviations are the same as in Supplementary Table 1.
