# Supplementary material for: Prognostic Value of Right Ventricular Strains Using Novel Three-Dimensional Analytical Software in Patients With Cardiac Disease
Source: Front Cardiovasc Med. 2022 Feb 25;9:837584. doi: 10.3389/fcvm.2022.837584 (PMC8914046; doi:10.3389/fcvm.2022.837584)
Supplement: Supplementary Table 3 — Clinical and echocardiography parameters in patients with and without HF hospitalization. Data are expressed as numbers (percentages) or medians [interquartile ranges]. NNT, number needed to treat. Other abbreviations are the same as in Supplementary Table 1. [file Table_3.docx]

**Supplementary Table 3: Clinical and echocardiography parameters in patients with and without HF hospitalization.**

|  | Overall (n=341) | HF (+) (n=37) | HF (-) (n=304) | P-value | NNT |
| --- | --- | --- | --- | --- | --- |
| Age (year) | 68 [58, 76] | 74 [65, 79] | 68 [57, 75] | 0.021 | 4.6 |
| Sex (male) (%) | 226 (66%) | 23 (62%) | 203 (67%) | 0.6 | 21.7 |
| BSA (/m^2^) | 1.62 [1.50, 1.75] | 1.59 [1.45, 1.75] | 1.62 [1.50, 1.75] | 0.4 | 9.4 |
| HT (%) | 191 (56%) | 24 (65%) | 167 (55%) | 0.3 | 10.1 |
| DM (%) | 101 (30%) | 13 (35%) | 88 (29%) | 0.4 | 16.2 |
| HL (%) | 149 (44%) | 16 (43%) | 133 (44%) | 0.9 | 197.3 |
| CAD (%) | 143 (42%) | 15 (41%) | 128 (42%) | 0.9 | 63.9 |
| CKD (%) | 149 (44%) | 23 (62%) | 126 (41%) | 0.016 | 4.8 |
| HR (beat/minute) | 67 (59, 76) | 70 [60, 82] | 66 [59, 75] | 0.2 | 6.9 |
| SBP (mmHg) | 127 [112, 145] | 117 [108, 134] | 129 [112, 145] | 0.069 | 6.2 |
| DBP (mmHg) | 71 [63, 79] | 67 [58, 75] | 72 [64, 80] | 0.011 | 3.6 |
| 3D LVEDVI (mL/m^2^) | 90 [71, 124] | 109 [79, 129] | 89 [71, 123] | 0.026 | 4.8 |
| 3D LVESVI (mL/m^2^) | 52 [36, 85] | 71 [50, 100] | 50 [35, 83] | 0.005 | 3.9 |
| 3D LVEF (%) | 41 [28, 50] | 31 [24, 42] | 43 [30, 51] | <0.001 | 3.0 |
| 3D LVGLS (%) | 12.2 [7.8, 15.5] | 8.0 [5.8, 11.6] | 12.5 [8.6, 15.8] | <0.001 | 2.7 |
| 3D LAVI max (mL/m^2^) | 48 [35, 66] | 56 [46, 64] | 37 [29, 52] | <0.001 | 2.6 |
| 3D LAVI min (mL/m^2^) | 31 [20, 47] | 43 [37, 52] | 25 [17, 40] | <0.001 | 2.4 |
| E (cm/sec) | 66 [49, 85] | 78 [62, 93] | 65 [49, 82] | 0.024 | 6.6 |
| A (cm/sec) | 70 [51, 90] | 76 [49, 92] | 70 [52, 89] | 0.8 | 12.7 |
| Average mitral E/e’ | 11.4 [8.4, 15.2] | 13.5 [10.6, 19.8] | 11.1 [8.2, 14.7] | 0.005 | 3.1 |
| SPAP (mmHg) | 31 [25, 38] | 38 [32, 43] | 31 [25, 37] | 0.011 | 3.4 |
| TAPSE (mm) | 16.7 [13, 20.6] | 14 [12, 18] | 17 [13, 21] | 0.009 | 4.2 |
| RV s’ (cm/sec) | 10.6 [8.8, 12.3] | 9.8 [8.8, 11.1] | 10.7 [8.8, 12.5] | 0.14 | 8.1 |
| TomTec |  |  |  |  |  |
| 3D RVEDVI (mL/m^2^) | 61 [51, 76] | 76 [58, 90] | 61 [50, 73] | <0.001 | 3.0 |
| 3D RVESVI (mL/m^2^) | 32 [25, 42] | 44 [35, 58] | 31 [24, 41] | <0.001 | 2.3 |
| 3D RVEF (%) | 48 [40, 54] | 40 [31, 46] | 49 [40, 54] | <0.001 | 2.2 |
| ReVISION |  |  |  |  |  |
| 3D RVEDVI (mL/m^2^) | 61 [51, 76] | 76 [58, 90] | 61 [50, 73] | <0.001 | 3.1 |
| 3D RVESVI (mL/m^2^) | 33 [25, 42] | 44 [35, 58] | 31 [24, 41] | <0.001 | 2.3 |
| 3D RVEF (%) | 47 [39, 54] | 40 [31, 46] | 48 [40, 54] | <0.001 | 2.2 |
| 3D RVGCS (%) | 19.5 [15.7, 23.3] | 15.9 [12.2, 20.2] | 20.0 [16.3, 23.7] | <0.001 | 2.6 |
| 3D RVGLS (%) | 15.2 [12.1, 18.4] | 12.4 [9.8, 14.6] | 15.6 [12.7, 18.8] | <0.001 | 2.6 |
| 3D RVGAS (%) | 30.0 [24.2, 35.4] | 24.9 [17.5, 29.9] | 30.4 [24.8, 35.7] | <0.001 | 2.4 |

Data are expressed as numbers (percentages) or medians [interquartile ranges].

NNT, number needed to treat. Other abbreviations are the same as in Supplementary Table 1.
