# Supplementary material for: Prognostic Value of Right Ventricular Strains Using Novel Three-Dimensional Analytical Software in Patients With Cardiac Disease
Source: Front Cardiovasc Med. 2022 Feb 25;9:837584. doi: 10.3389/fcvm.2022.837584 (PMC8914046; doi:10.3389/fcvm.2022.837584)
Supplement: Supplementary Table 4 — Univariate Cox proportional hazard analysis for HF hospitalization. Abbreviations are the same as in Supplementary Table 1. [file Table_4.docx]

**Supplementary Table 4: Univariate Cox proportional hazard analysis for HF hospitalization**

| Variables | HR | 95% CI | P-value |
| --- | --- | --- | --- |
| Age (year) | 1.04 | 1.01 to 1.07 | 0.009 |
| Gender (male) | 0.96 | 0.49 to 1.88 | 0.9 |
| BSA (/m^2^) | 0.51 | 0.10 to 2.56 | 0.4 |
| HR (beat/min) | 1.02 | 1.00 to 1.04 | 0.11 |
| SBP (mmHg) | 0.99 | 0.98 to 1.01 | 0.2 |
| DBP (mmHg) | 0.96 | 0.93 to 0.99 | 0.009 |
| HT (%) | 1.57 | 0.80 to 3.08 | 0.2 |
| DM (%) | 1.29 | 0.65 to 2.53 | 0.5 |
| HL (%) | 0.96 | 0.50 to 1.83 | 0.9 |
| CAD (%) | 1.02 | 0.53 to 1.97 | 0.9 |
| CKD (%) | 3.17 | 1.61 to 6.24 | <0.001 |
| 3D LVEDVI (mL/m^2^) | 1.01 | 1.00 to 1.01 | 0.086 |
| 3D LVESVI (mL/m^2^) | 1.01 | 1.00 to 1.02 | 0.021 |
| 3D LVEF (%) | 0.96 | 0.94 to 0.98 | <0.001 |
| 3D LVGLS (%) | 0.88 | 0.82 to 0.94 | <0.001 |
| 3D LAVI max (mL/m^2^) | 1.02 | 1.01 to 1.03 | <0.001 |
| 3D LAVI min (mL/m^2^) | 1.02 | 1.01 to 1.03 | <0.001 |
| E wave (cm/sec) | 1.00 | 0.99 to 1.01 | 0.4 |
| A wave (cm/sec) | 1.00 | 0.99 to 1.02 | 0.5 |
| Average mitral E/e’ | 1.07 | 1.02 to 1.11 | 0.002 |
| SPAP (mmHg) | 1.03 | 1.01 to 1.06 | 0.004 |
| TAPSE (mm) | 0.91 | 0.86 to 0.98 | 0.007 |
| RV s’ (cm/sec) | 0.91 | 0.79 to 1.04 | 0.2 |
| TomTec |  |  |  |
| 3D RVEDVI (mL/m^2^) | 1.02 | 1.01 to 1.03 | 0.003 |
| 3D RVESVI (mL/m^2^) | 1.03 | 1.01 to 1.04 | <0.001 |
| 3D RVEF (%) | 0.93 | 0.90 to 0.96 | <0.001 |
| ReVISION |  |  |  |
| 3D RVEDVI (mL/m^2^) | 1.02 | 1.01 to 1.03 | 0.003 |
| 3D RVESVI (mL/m^2^) | 1.03 | 1.01 to 1.04 | <0.001 |
| 3D RVEF (%) | 0.93 | 0.90 to 0.96 | <0.001 |
| 3D RVGCS (%) | 0.88 | 0.83 to 0.94 | <0.001 |
| 3D RVGLS (%) | 0.84 | 0.78 to 0.91 | <0.001 |
| 3D RVGAS (%) | 0.91 | 0.87 to 0.95 | <0.001 |

Abbreviations are the same as in Supplementary Table 1.
