# Supplementary material for: Prognostic Value of Right Ventricular Strains Using Novel Three-Dimensional Analytical Software in Patients With Cardiac Disease
Source: Front Cardiovasc Med. 2022 Feb 25;9:837584. doi: 10.3389/fcvm.2022.837584 (PMC8914046; doi:10.3389/fcvm.2022.837584)
Supplement: Supplementary Table 5 — Result of reproducibility analysis for right ventricular volumes and right ventricular ejection fraction. ICC, intraclass correlation coefficients; IQR, interquartile ranges. Other abbreviations are the same as in Supplementary Table 1. [file Table_5.docx]

**Supplementary Table 5: Result of reproducibility analysis for right ventricular volumes and right ventricular ejection fraction.**

|  | Intra-observer variability | | Inter-observer variability | |
| --- | --- | --- | --- | --- |
|  | % variability  median (IQR) | ICC | % variability  median (IQR) | ICC |
| RVEDV | 4.5 (1.5-8.4) | 0.97 | 7.4 (1.7-13.6) | 0.93 |
| RVESV | 5.4 (2.5-11.2) | 0.95 | 7.4 (3.9-16.4) | 0.91 |
| RVEF | 5.4 (2.0-11.1) | 0.92 | 9.2 (6.4-11.7) | 0.86 |

ICC, intraclass correlation coefficients; IQR, interquartile ranges. Other abbreviations are the same as in Supplementary Table 1.
